# Clinical study on the impact of microwave ablation energy on the treatment efficacy of benign thyroid nodules

**DOI:** 10.3389/fonc.2025.1568697

**Published:** 2025-07-18

**Authors:** Xue Mei, Qianqian Fu, Guangyin Li, Xin Ai, Yu Sun, Jiawei Tian, Xiaoping Leng, Shuangquan Jiang

**Affiliations:** ^1^ Department of Ultrasound Medicine, The Second Affiliated Hospital of Harbin Medical University, Harbin, Heilongjiang, China; ^2^ Department of Ultrasound Medicine, Harbin Medical University Cancer Hospital, Harbin, Heilongjiang, China; ^3^ Daqing People’s Hospital, Ultrasound Room, Daqing, Heilongjiang, China; ^4^ Department of Thyroid Surgery, The Second Affiliated Hospital of Harbin Medical University, Harbin, Heilongjiang, China

**Keywords:** thyroid nodule, ablation techniques, microwaves, energy transfer, ultrasonography

## Abstract

**Objective:**

We aimed to establish a model to estimate the energy required for microwave ablation (MWA) to achieve the desired effect and analyze the factors influencing its therapeutic efficacy.

**Materials and Methods:**

We retrospectively analyzed 117 patients with benign thyroid nodules. A quadratic regression model was established to analyze the relationship between the technical parameters of MWA and volume reduction rate (VRR). Both univariate and multivariate logistic regression analyses were used to identify factors influencing the efficacy of MWA treatment.

**Results:**

The volume of nodules continued to decrease at 1, 3, 6, and 12 months after ablation, and the mean of VRR was 77.5 ± 15.9% at 12 months after ablation. Among these nodules, 72 (61.5%) had a VRR ≥ 75%, whereas 45 (38.5%) had a VRR < 75%. The energy volume ratio was significantly correlated with the VRR. When the VRR ≥ 75%, the energy volume ratio ranges 784-2,274 J/mL. Among all parameters, only the energy volume ratio and calcification were independent factors influencing the treatment efficacy for benign thyroid nodules (*P* < 0.05).

**Conclusions:**

The efficacy of the treatment was optimized when the energy volume ratio of the MWA fell within a certain range. The energy volume ratio and calcification are related to the efficacy of MWA treatment.

## 1 Introduction

The proportion of benign thyroid nodules is 80–95%, and clinical intervention is required when the nodules are large, have local compression symptoms, or affect appearance (1). Thermal ablation has recently become a new treatment option for thyroid nodules. Ultrasound-guided thermal ablation has the advantages of less trauma, faster recovery, high safety, and no influence on thyroid function. Several guidelines recommend it as an alternative to surgical treatment or regular observation (2, 3). Numerous studies have revealed that thermal ablation has favorable effects on benign thyroid nodules (4–6). Liu et al. used microwave ablation (MWA) to treat 173 cases of benign thyroid nodules, and the volume reduction ratio (VRR) of the nodules reached 89% 1 year after treatment (7). Cappelli et al. used radiofrequency ablation (RFA) to treat functional and nonfunctional thyroid nodules, and the average VRR of nodules was 69.1 ± 17.3% at 1 year after treatment (8). Cesareo et al. compared the efficacy of RFA and laser ablation (LA) in the treatment of benign thyroid nodules, and the VRR was 70.9 ± 16.9% and 60.0 ± 19.0% 1 year after treatment, respectively (9).

There are significant differences in the therapeutic efficacy of different study populations and thermal ablation treatment methods. In recent years, a few researchers have attempted to explore the influencing factors of the efficacy of thermal ablation therapy. Deandrea et al. revealed that nodule composition, initial volume, and blood flow distribution are related to postoperative efficacy (10). Fu et al. revealed that the internal components of the nodules, enhancement pattern, and immediate postoperative volume were independent factors that influenced the curative effect (11). Li et al. showed that the VRR of nodules in a group with coarse calcifications was lower (12). Numerous studies have been conducted on the effects of nodule characteristics on the efficacy of ablation (13–15). In addition, the influence of technical parameters of thermal ablation on its effectiveness has also been reported. Trimboli et al. discussed the technical parameters related to RFA in the treatment of benign thyroid nodules, and their results revealed that the energy volume ratio was significantly correlated with the VRR (16). Gambelunghe et al. evaluated the effect of the energy volume ratio on the efficacy of LA in the treatment of benign thyroid nodules and found that the treatment was effective only when the energy volume ratio was > 400–500 J/mL (17). Amabile et al. found that lower LA energy significantly reduced the volume of benign thyroid nodules (18). At present, research on the influence of the technical parameters of thermal ablation on the therapeutic effects mostly focuses on LA and RFA. However, a few studies report on the effects of the technical parameters of MWA on therapeutic efficacy. Because the working principle of MWA is different from that of LA and RFA, the results may also be different. Therefore, we aimed to explore the parameters that affect the therapeutic effects of MWA.

## 2 Materials and methods

### 2.1 Participants

We retrospectively analyzed 307 patients treated with ultrasound-guided MWA at our hospital between September 2021 and December 2023. Of these, 190 were excluded due to loss to follow-up or incomplete data during follow-up, and 117 patients, including 10 males and 107 females, were included. The inclusion criteria were as follows: (1) fine needle aspiration or core needle biopsy confirmed that the nodule was benign; (2) unilateral single solid nodule with a maximum diameter of ≥ 2 cm and increasing gradually (19); (3) presence of symptoms such as neck compression, foreign body sensation, or patients actively requesting treatment due to anxiety. The exclusion criteria were as follows: (1) previous thyroid surgery or ablation therapy; (2) severe heart and lung disease or coagulation dysfunction; and (3) incomplete clinical, ultrasound, and pathological data. This study was approved by the Academy Ethics Committee (Ethics Number: YJSKY2022-399).

### 2.2 Evaluation prior to MWA

An Aixplorer ultrasound system (SuperSonic Image. SA, France) equipped with an L15–4 high-frequency linear probe (4–15MHz) and L10–2 high-frequency linear probe (2–10MHz) was used for the evaluation and ablation treatment guidance of nodules. The ablation instrument used was the KY2000 microwave ablation therapy instrument (Nanjing Kangyou Medical Technology Company Limited) equipped with a 16G sterile disposable microwave ablation needle.

The size, echo, calcification, and blood flow distribution of the nodules were evaluated using ultrasonography before MWA. According to the diameter of the three dimensions of the nodule, the nodule volume was calculated in the following manner: V = a × b × c × Π/6 (V: volume, a: upper and lower diameter, b: left and right diameter, c: anterior and posterior diameter). The patient underwent electrocardiography, laryngoscopy and laboratory tests before ablation. Before treatment, patients and their families signed an informed consent form.

### 2.3 MWA treatment process and technical parameters

MWA was performed after a fine preoperative evaluation. Vital signs including blood pressure, heart rate, and blood oxygen levels were continuously monitored during the operation. The surgical area was disinfected, the hole towel was spread, the optimal puncture site was determined under ultrasound guidance in real time, and 5 mL of 2% lidocaine was injected subcutaneously for local infiltration anesthesia. Further, 20–40 mL of normal saline and lidocaine mixed liquid (18:2) is injected between the thyroid gland and adjacent important tissues (such as common carotid artery, recurrent laryngeal nerve, trachea, esophagus, etc.) to provide an “isolation zone” for ablation treatment and avoid damage to surrounding tissues. According to the specific location of the nodule, a translateral cervical or transisthmus approach was used, and moving-shot technique was used to ablate the target nodule layer-by-layer. The ablation needle was withdrawn when the target nodule was completely covered by the strong-echo gasification zone and CDFI showed no blood flow signal within the nodule. Contrast-enhanced ultrasound examination was performed immediately after the pneumatization subsided to evaluate whether the ablation treatment was complete, and additional ablation was performed on the area with residual contrast agent perfusion inside the nodule. During the ablation process, the patient’s feelings were intermittently investigated, and whether the patient experienced thermal damage to the recurrent laryngeal nerve was assessed based on vocalization. The mobility of the false vocal cords was also evaluated using ultrasonography. After the ablation treatment was completed, cold compression was applied to the ablation area for 1 h to stop bleeding and reduce pain. Patients were observed for 4 h after the operation, and the occurrence of complications was recorded in detail. All patients were treated by the same surgeon with > 8 years of ablation experience.

The output power of the microwave ablation instrument was set to 30 W, the duration of MWA treatment was recorded, and the total ablation energy was expressed in joules (total ablation energy = ablation time × ablation power). The energy volume ratio was calculated using the following formula: energy volume ratio = total ablation energy/nodule volume.

### 2.4 Postoperative follow-up

Thyroid ultrasound examination was performed 1, 3, 6, and 12 months after ablation to observe blood perfusion in the nodules. The VRR of the nodule was calculated as follows: VRR = (baseline volume – final volume)/baseline volume × 100%. With 12 months after ablation as the observation endpoint, the patients were classified into the significantly effective group (VRR ≥ 75%) and the nonsignificantly effective group (VRR < 75%) based on whether the VRR exceeded 75%.

### 2.5 Statistical analysis

SPSS26.0 software was used for data analysis. A quadratic regression model was used to analyze the relationship between the technical parameters of the MWA treatment and the VRR. Count data were expressed in rate (%), and χ^2^ test was used for comparison between groups. The normality test was performed for the continuous data, and those who conformed to the normal distribution were expressed by the mean ± standard deviation, and the independent samples t-test was used for comparison between groups. Continuous data that did not conform to a normal distribution were expressed as medians and upper and lower quartiles, and rank-sum tests were used for comparisons between the groups. Statistically significant factors in the univariate analysis were included in the binary logistic regression analysis. *P* < 0.05 was considered statistically significant.

## 3 Results

**Figure 1:**
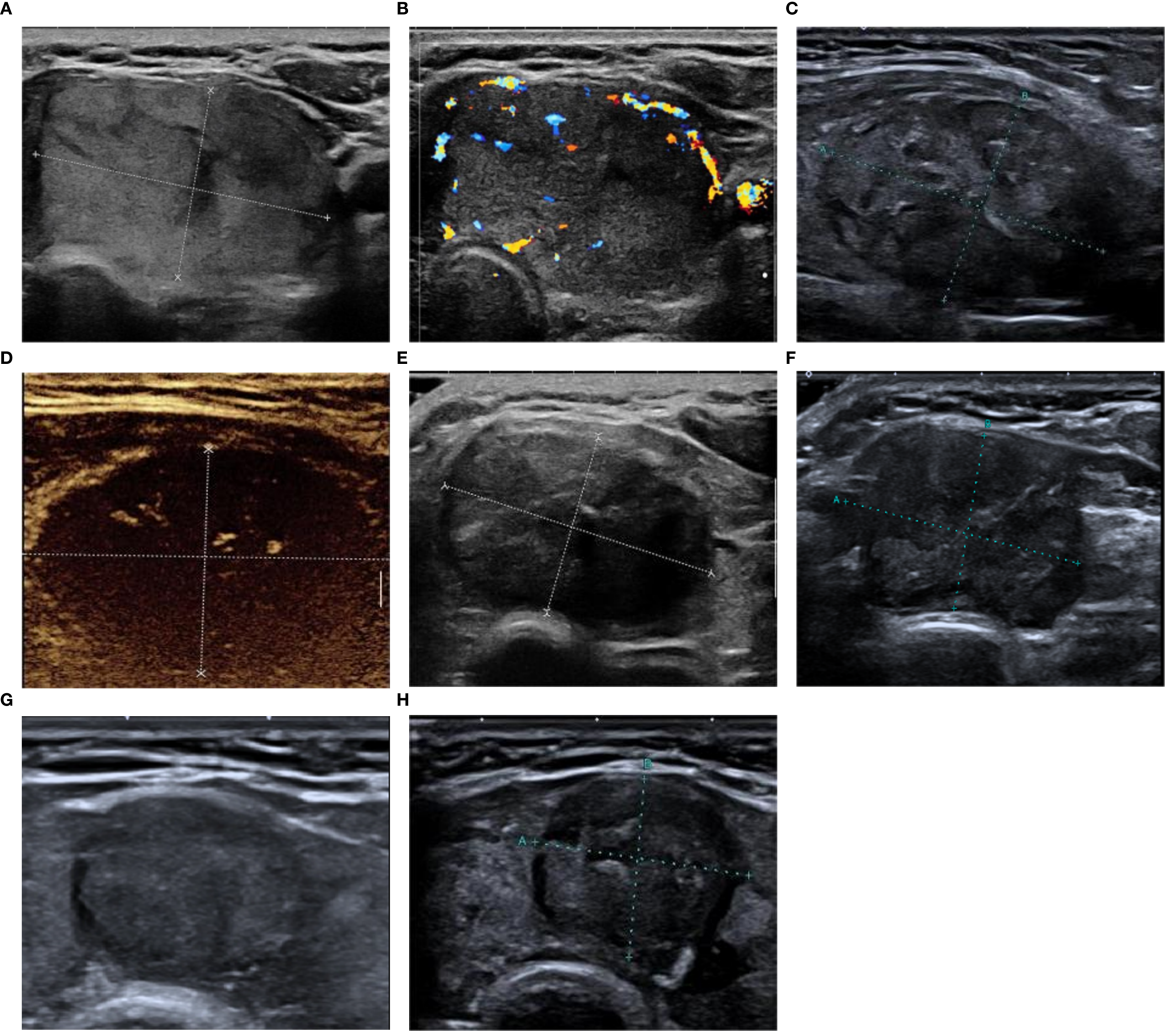
The patient is a 49-year-old female. **(A, B)** Preoperative ultrasound reveals large nodules in the thyroid isthmus and left lobe, which are isoechoic, with clear borders, Adler blood flow grade III, no calcification, size 3.92 cm × 2.05 cm × 4.04 cm, and volume 16.9 mL; **(C)** Two-dimensional ultrasound immediately after MWA reveals an oval-shaped hypoechoic state with clear boundaries; **(D)** Contrast-enhanced ultrasound examination immediately after MWA reveals no perfusion within the nodule; **(E-H)** Two-dimensional ultrasound images obtained at 1, 3, 6, and 12 months after treatment, respectively. The lesion volume seems to have decreased gradually, and VRR is 78.5% at 12 months after ablation. MWA, microwave ablation; VRR, volume reduction rate.

All 117 patients completed postoperative follow-up at each time point, and the nodule volume gradually decreased (**Figure 1**). None of the patients experienced tumor recurrence or regeneration. At 12 months after the operation, the mean VRR of nodules was 77.5 ± 15.9% (29.1%-98.6%). Among them, 72 cases (61.5%) had a VRR ≥ 75%, and 45 cases (38.5%) had a VRR < 75%. Eight patients (6.8%) experienced side effects after ablation, including seven cases of neck pain and one case of fever. The symptoms disappeared within 1 day to 1 week of ablation without treatment. Two patients (1.7%) experienced minor complications of hoarseness, which returned to normal during the follow-up examination 1 month after treatment.

### 3.1 Changes in the thyroid nodule volume after MWA treatment

**Figure 2:**
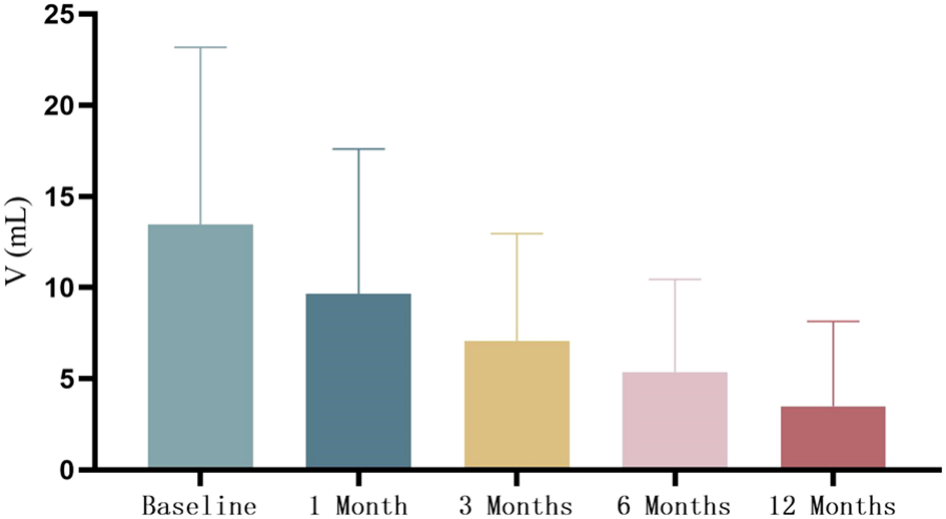
Volume comparison before and after MWA. MWA, microwave ablation.

The mean volume of thyroid nodules was (13.47 ± 9.72) mL before MWA, and the volume of thyroid nodules continued to shrink at 1, 3, 6, and 12 months after operation, which were 9.66 ± 7.94, 7.08 ± 5.89, 5.37 ± 5.07, 3.49 ± 4.65 mL, respectively (**Figure 2**). The average VRRs increased gradually at 1, 3, 6, and 12 months after operation, which were 28.9 ± 18.4%, 47.0 ± 16.9%, 61.2 ± 16.0%, 77.5 ± 15.9%, respectively.

**Figure 3:**
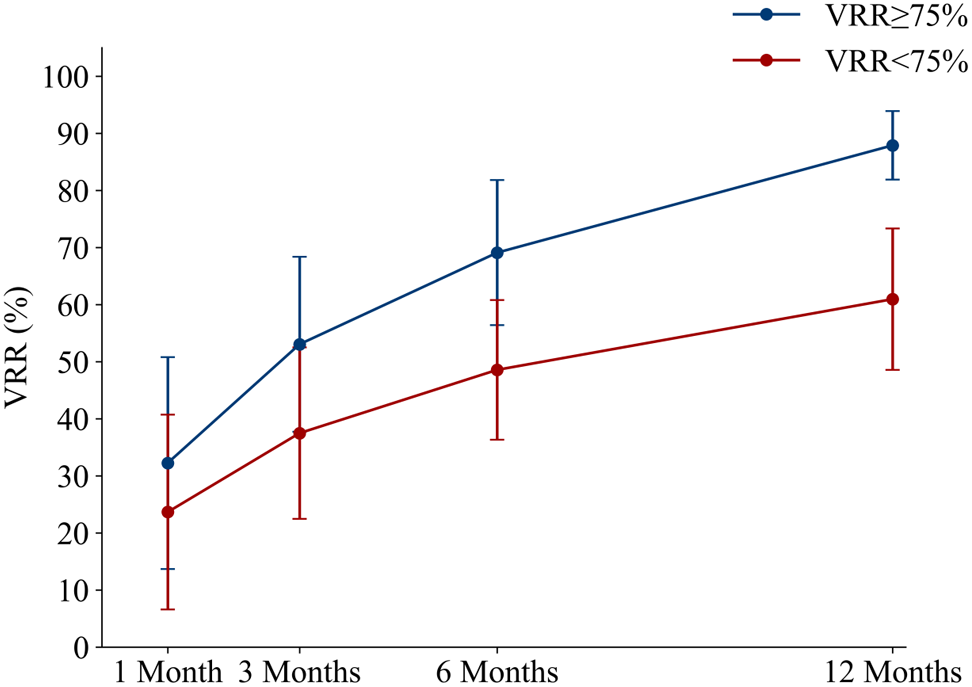
The average volume reduction rate of patients in the two groups at each follow-up time point after MWA. MWA, microwave ablation.

The VRR of the two groups at different time points after the operation were compared using a repeated-measures analysis of variance. The results showed significant differences in the VRR between the two groups at different observation time points (F = 455.48, *P* < 0.01). In addition, at the same observation time point, the difference in VRR between the two groups was statistically significant (F = 69.91, *P* < 0.01). (**Table 1**, **Figure 3**).

**Table 1:** Comparison of VRR of nodules at various follow-up time points after operation.

	1 month	3 months	6 months	12 months
VRR ≥ 75% (%)	32.24 ± 18.56	53.03 ± 15.32	69.09 ± 12.71	87.88 ± 6.02
VRR < 75% (%)	23.64 ± 17.06	37.46 ± 15.02	48.56 ± 12.23	60.94 ± 12.40

### 3.2 Relationship between technical parameters and efficacy of MWA treatment

**Figure 4:**
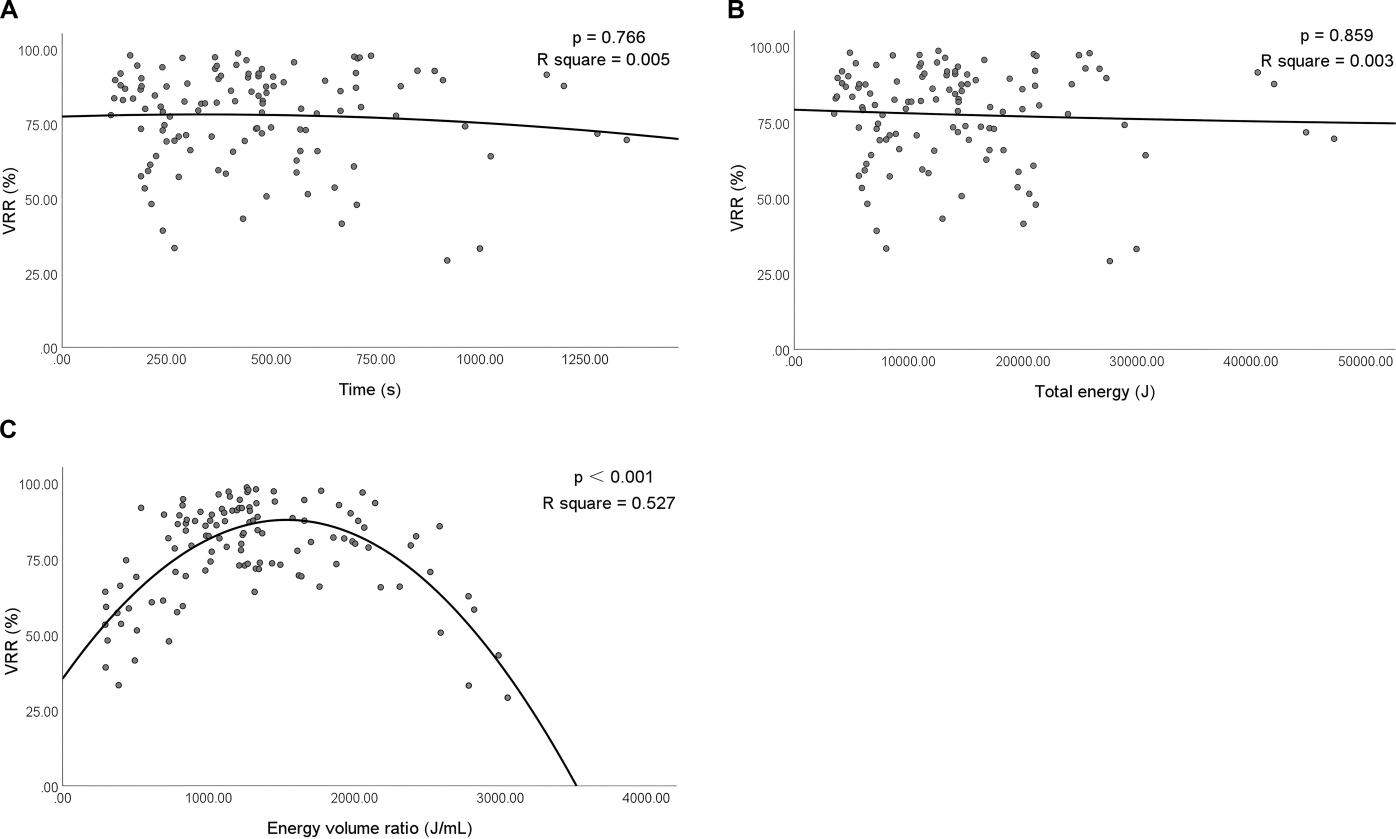
Correlation among the ablation time, total ablation energy, energy volume ratio, and VRR at 12 months after ablation. VRR, volume reduction rate. P<0.001 was considered statistically significant. R square represents the proportion of the variance for a dependent variable that's explained by the independent variables in the regression model.

A quadratic regression model was established to analyze the relationship between the ablation time, total energy, energy volume ratio, and VRR. The results revealed that only the energy volume ratio was significantly correlated with VRR at 12 months after ablation (*P* < 0.001), and the ablation time and total ablation energy were not related to VRR (*P* = 0.766 and *P* = 0.859). According to the fitting curve of the established model, the energy volume ratio was between 784 J/mL and 2274 J/mL, when the VRR ≥ 75% (**Figure 4**).

**Figure 5:**
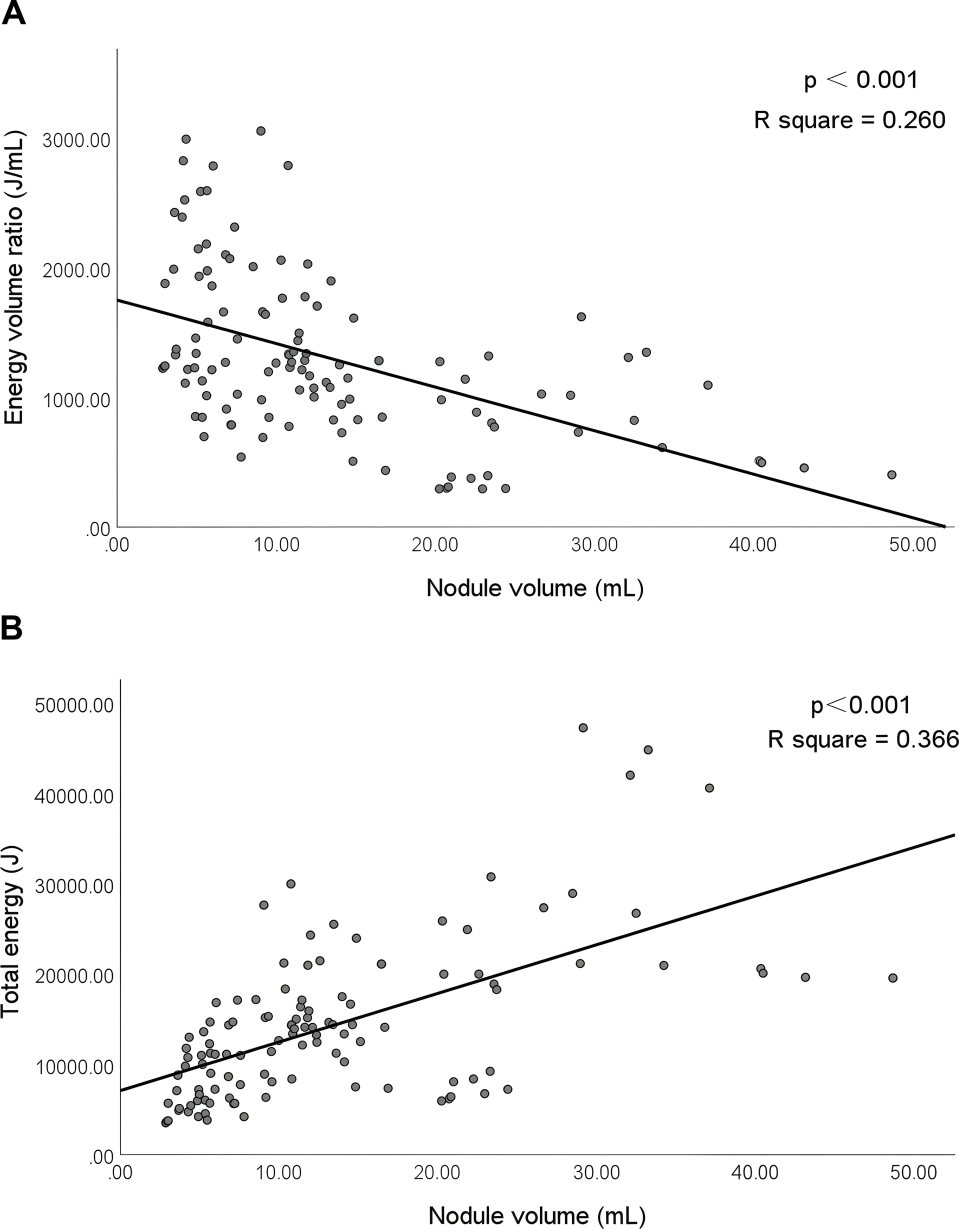
Correlation between the energy volume ratio, total energy, and nodule volume. P<0.001 was considered statistically significant. R square represents the proportion of the variance for a dependent variable that's explained by the independent variables in the regression model.

The collinearity between the energy volume ratio, total energy, and nodule volume was analyzed. The results showed that the energy volume ratio and total energy were related to the nodule volume (both *P* < 0.001) (**Figure 5**).

### 3.3 Influencing factors of MWA treatment efficacy

Univariate analysis was performed on general clinical data, ultrasound characteristics, and technical parameters related to MWA treatment. The results revealed significant differences in nodule volume, echo, calcification, and energy volume ratio between the two groups (all *P* < 0.05) (**Table 2**). Multivariate logistic regression analysis was performed on the four statistically significant variables, and the results revealed that calcification and energy volume ratio were independent influencing factors for the treatment efficacy of benign thyroid nodules (all *P* < 0.05) (**Table 3**).

**Table 2:** Analysis of influencing factors for the efficacy of benign thyroid nodules.

Factors	VRR ≥ 75% (*n* = 72)	VRR < 75% (*n* = 45)	χ^2^/t/Z	*P* value
Gender			0.011	0.917
Male	6 (60.0%)	4 (40.0%)		
Female	66 (61.7%)	41 (38.3%)		
Age(years)	43.93 ± 12.27	43.98 ± 13.13	0.020	0.984
Maximum diameter(cm)	3.75 ± 0.73	4.05 ± 0.94	1.904	0.059
Nodule volume			10.371	0.001
≥ 20mL	10 (35.7%)	18 (64.3%)		
< 20mL	62 (69.7%)	27 (30.3%)		
Echo			5.265	0.022
(Extreme) low echo	46 (70.8%)	19 (29.2%)		
Non-low echo	26 (50.0%)	26 (50.0%)		
Calcification			—	<0.001^*^
No	69 (70.4%)	29 (29.6%)		
Yes	3 (15.8%)	16 (84.2%)		
Adler blood flow grading			1.483	0.686
Level 0	3 (75.0%)	1 (25.0%)		
Level I	12 (52.2%)	11 (47.8%)		
Level II	27 (65.9%)	14 (34.1%)		
Level III	30 (61.2%)	19 (38.8%)		
Ablation time(s)	478.0 (370.0–701.0)	437.0 (259.5–599.5)	-0.647	0.518
Total energy(J)	14340.0 (11100.0–21030.0)	13920.0 (7785.0–19597.5)	-0.913	0.361
Energy volume ratio			—	<0.001^*^
784-2274J/mL	65 (77.4%)	19 (22.6%)		
<784J/mL or >2274J/mL	7 (21.2%)	26 (78.8%)		

*Fisher exact probability method was used.

**Table 3:** Multivariate logistic regression analysis affecting the efficacy of benign thyroid nodules.

Factors	*β*	SE	Wald	*P*	*OR*	95%*CI*
Nodule volume	-0.685	0.559	1.501	0.221	0.504	0.169, 1.508
Echo	-0.270	0.480	0.316	0.574	0.764	0.298, 1.956
Calcification	1.649	0.742	4.934	0.026	5.201	1.214, 22.280
Energy volume ratio	1.972	0.540	13.332	<0.001	7.184	2.493, 20.704

## 4 Discussion

The incidence of thyroid nodules is increasing year by year, with a detection rate of 65% in the population (20). Most benign thyroid nodules required follow-up. Large thyroid nodules can cause varying degrees of dyspnea or swallowing discomfort and require aggressive management. In 2000, LA was first applied for the palliative treatment of recurrent thyroid cancer, leading to an era of minimally invasive thermal ablation therapy for thyroid nodules (21). In 2006, Kim applied RFA to the ablation treatment of benign thyroid nodules, thereby confirming the feasibility of RFA for the treatment of benign thyroid nodules (22). In 2012, Feng performed MWA treatment for 11 benign thyroid nodules for the first time, and the VRR was 45.99 ± 29.90% at 12 months after treatment and the related symptoms were found to be improved (23). Over the last 20 years, patients and clinicians have gradually recognized the need for ultrasound-guided thermal ablation therapy. Compared with lasers and radiofrequency, MWA has the advantages of fast heating and a large ablation range and is more suitable for the treatment of larger nodules. In this study, 117 patients with benign thyroid nodules were treated with ultrasound-guided MWA. The characteristics of the nodules were evaluated by two-dimensional ultrasound before MWA. Elastography was used to assist in determining the benign or malignant nature of the nodules (24–26). Additionally, a fine-needle aspiration biopsy or core needle aspiration was performed to confirm the nature of the nodules. The results revealed that with the extension of the follow-up time, the volume of thyroid nodules gradually decreased, and the VRR gradually increased. Twelve months after ablation, 109 patients (93.2%) had a VRR ≥ 50%, 72 patients (61.5%) had a VRR ≥ 75%, and 29 patients (24.8%) had a VRR ≥ 90%. After treatment, a small number of patients experienced side effects, such as neck pain and fever, and the symptoms disappeared quickly. Only two patients (1.7%) had mild complications of hoarseness, which recovered during the follow-up examination 1 month after ablation. Postoperative laryngoscopy was performed to determine whether vocal cord movement was normal. The results showed that MWA treatment of benign thyroid nodules was safe and effective.

Univariate analysis revealed a statistically significant difference in nodule size between the two groups. This study was based on whether the nodule volume was ≥ 20mL. The results showed that smaller nodules had a higher VRR. This is consistent with the results of Dobnig et al. (27), who conducted a retrospective analysis of 154 benign thyroid nodules treated with RFA that revealed a VRR of 82 ± 13% at 12 months postoperatively. According to the initial nodule volume, the VRR was higher in patients with smaller initial volumes. Previous studies also concluded that the VRR of small nodules was more significant after MWA treatment (11), which may be because the larger the nodules, the more necrotic the tissue after ablation, and the longer it takes for the body to phagocytose and clear (28). Nodule echo can also affect the therapeutic effect of MWA, and (extremely) low-echo nodule VRR was higher than that of nonlow-echo VRR, and the therapeutic effect was more significant. This is consistent with previous studies, and Cao explored the independent influencing factors of the VRR after thermal ablation of benign thyroid nodules, which also revealed that low-echo nodules had a higher VRR (29). Kim et al. concluded that low-echo thyroid nodules contain more cells and fibrous tissue (30). The absorption of the ablation area is mainly dependent on the immune response caused by cell necrosis after thermal ablation. In low-echo nodules, more cells were absorbed more easily.

Furthermore, a statistically significant difference was observed in calcification between the two groups, and the results of multivariate logistic regression analysis revealed that calcification was an independent influencing factor affecting the efficacy of MWA treatment for benign thyroid nodules. At 12 months after ablation, noncalcified nodules were more effective than those with calcification, with VRR ≥ 75% in 70.4% of noncalcified nodules and 15.8% of those with calcification. This is consistent with the findings of He et al. (31), who retrospectively analyzed 204 cases of papillary thyroid carcinoma treated with RFA and constructed a nomogram to predict whether the ablation area could be fully absorbed. These results reveal that calcification is closely related to whether the ablation area is fully absorbed. This may be due to the fact that the calcification impairs the conduction of heat energy and impairs the body’s absorption of the ablation area.

In addition to the influence of nodule characteristics on the curative effect, we conducted an in-depth analysis of the influence of MWA technical parameters on the curative effect. Currently, there are few reports on the relationship between the technical parameters of thermal ablation and its therapeutic effect. Gambelunghe et al. (17) evaluated the technical parameters of the therapeutic effect of LA on benign thyroid nodules and found that treatment was effective only when the energy volume ratio was > 400–500 J/mL. Deandrea et al. (32) conducted a regression analysis on the energy required by RFA for the treatment of benign thyroid nodules and found that when the nodules were administered 756 J/mL, the treatment was effective with a 50% probability, and when the nodules were administered 2670 J/mL, the treatment was effective with a 99% probability. However, there are a few studies on the effect of MWA energy on the efficacy of treatment, and the results are not consistent. Korkusuz et al. (33) studied the energy required by MWA to treat symptomatic benign thyroid nodules, and their results revealed that the energy delivered by ablation was positively correlated with volume reduction. Cao et al. (29) showed that the VRR ≥ 75% group required a lower energy volume ratio than the VRR < 75% group, based on whether the VRR was ≥ 75% after MWA.

This study explored the relationship between the MWA time, total energy, energy volume ratio, and VRR. Through the establishment of a quadratic regression model, the results revealed that only the energy volume ratio was correlated with the VRR, and the fitting curve was parabolic. According to the fitting curve of the established model, when the energy volume ratio was between 784 and 2274 J/mL, the curative effect was found to be better; thus, the VRR could achieve more ideal results. The ablation time and total ablation energy were not related to the VRR. Multivariate logistic regression analysis also confirmed that the energy volume ratio was an independent factor affecting the VRR. The different results obtained using different thermal ablation methods may be related to their different working principles. MWA transmits electromagnetic energy through the antenna; polar water molecules rotate and function at a high speed under the action of the electric field, and friction generates heat, which, in turn, increases the temperature of the tissue, leading to coagulation necrosis of tumor tissues in the target area, thereby resulting in *in-situ* inactivation of the tumor. Subsequently, necrotic tissue is engulfed by macrophages and the tumor constantly shrinks and disappears (34). Therefore, in the process of ablation therapy, if the energy volume ratio is insufficient, tissue cell inactivation will be incomplete; if the ablation time is too long, the temperature of the tissue center will be too high and carbonization will occur, thereby resulting in postoperative malabsorption. This may explain why the relationship between the energy volume ratio and VRR is parabolic. The energy volume ratio is negatively correlated with nodule volume, which may be related to the moving-shot technique adopted for thyroid nodules. During the ablation process of large nodules, high heat is maintained continuously when the microwave antenna retreats. This can relatively reduce the frequency of microwave antenna reboots. RFA differs from MWA in that the radiofrequency ablation instrument reads the real-time impedance directly related to tissue coagulation and necrosis; the longer the tissue coagulation time, the higher the impedance (35). When the impedance was increased to a certain level, the RFA generator did not transmit energy, thereby avoiding tissue carbonization. LA transmits photon energy to the optical fiber to generate heat and make the tissue coagulate necrosis to achieve the purpose of killing the tumor (36). Additionally, LA has the advantage of accurate positioning. However, its therapeutic power is low and the ablation range of a single needle is small; therefore, the treatment is effective only when the energy of the LA for treating benign thyroid nodules attains sufficient energy levels.

This study obtained technical parameters related to the efficacy of MWA by establishing a model. In addition, the energy required to achieve good results was obtained. However, this study has several limitations. First, this was a retrospective study, and there may have been some bias in the results. In addition, because of the large number of patients lost to follow-up, approximately 61.9% of the initially enrolled patients were eventually excluded from the study, thereby resulting in a slightly small sample size, which may have affected the reliability of the study results. Furthermore, the follow-up period in this study was relatively short, and the results after long-term follow-up are worth investigating. This is a direction for future research.

## 5 Conclusion

In conclusion, MWA is safe and effective for the treatment of benign thyroid nodules, with no major complications occurred. Energy volume ratio and calcification were independent factors that influenced the therapeutic effects of MWA. When the energy volume ratio of MWA is within a certain range, a better therapeutic effect can be achieved.

## Data Availability

The raw data supporting the conclusions of this article will be made available by the authors, without undue reservation.
